# Subperiosteal Excision of a Fibrolipoma in the Mandibular Alveolar Region Near the Mental Foramen: A Case Report

**DOI:** 10.7759/cureus.93388

**Published:** 2025-09-28

**Authors:** Yukio Watabe, Saimon Yasuhara, Shiro Shigematsu

**Affiliations:** 1 Department of Dentistry and Oral Surgery, Tokyo Metropolitan Tama Medical Center, Tokyo, JPN

**Keywords:** intraoral fibrolipoma, mandibular alveolar mucosa, mental foramen, neurovascular preservation, subperiosteal excision

## Abstract

Fibrolipomas of the oral cavity are uncommon, and occurrence in the mandibular alveolar mucosa adjacent to the mental foramen is particularly rare. We report a 68-year-old woman who presented with a painless swelling of the right lower gingiva. Clinical and radiologic evaluation identified a well-circumscribed lipomatous lesion located 3 mm anterior to the right mental foramen. Computed tomography (CT) demonstrated a homogeneous low-density mass, and magnetic resonance imaging (MRI) showed high signal intensity on T1- and T2-weighted sequences with signal suppression on short Tau inversion recovery (STIR), findings consistent with a lipomatous tumor. Given the lesion’s close relationship to the mental neurovascular bundle, complete excision was performed via a subperiosteal approach to preserve the overlying mucosa and minimize risk to sensory function. Under local anesthesia, a triangular mucoperiosteal flap was elevated, and the tumor was removed en bloc. Intraoperatively, no adhesion to the mental nerve was observed. Histopathology revealed mature adipocytes embedded in a dense fibrous stroma, confirming the diagnosis of fibrolipoma. The postoperative course was uneventful, with no sensory deficits or recurrence during follow-up. This case underscores two clinical points. First, fibrolipoma in this location is rare and requires thorough imaging to distinguish it from other submucosal masses. Second, a subperiosteal approach permits complete resection while maintaining mucosal integrity and protecting adjacent neurovascular structures, thereby reducing postoperative morbidity. Our experience reinforces the importance of tailoring surgical strategy to the lesion’s anatomy in intraoral lipomatous tumors.

## Introduction

Lipomas are benign mesenchymal tumors composed of mature adipocytes, most commonly found in the trunk, shoulders, and extremities. Although frequent in the general population, their occurrence in the oral cavity is rare, comprising 0.1-5% of all benign oral tumors [1]. Common intraoral locations include the buccal mucosa, lips, tongue, and floor of the mouth, while the mandibular alveolar mucosa is an exceptionally rare site [2-5].

Oral lipomas are often asymptomatic, slow-growing, and present as well-circumscribed, soft submucosal nodules. Imaging modalities, such as computed tomography (CT) and magnetic resonance imaging (MRI), provide critical diagnostic support in differentiating lipomatous lesions [6-8]. Surgical excision is the treatment of choice; however, in regions such as the mental foramen, meticulous planning is necessary to preserve nerve function and esthetic outcomes [9,10].

Here, we report a rare case of a fibrolipoma located in the mandibular alveolar region, 3 mm anterior to the mental foramen. The lesion was excised via a subperiosteal, mucosa-preserving approach using a triangular flap design. This case contributes valuable insights into both the rarity of the site and the advantages of a carefully planned surgical technique.

## Case presentation

**Figure 1:**
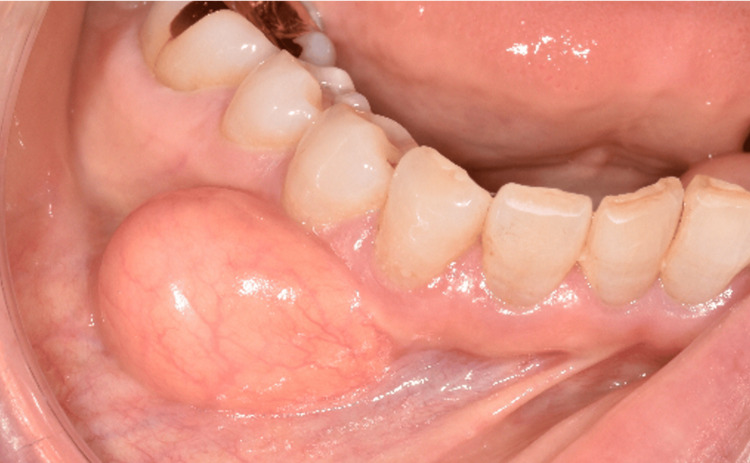
Clinical appearance of a submucosal mass in the right lower mandibular alveolar mucosa Intraoral preoperative photograph showing a submucosal swelling on the buccal alveolar mucosa between the right mandibular canine and first premolar. The overlying mucosa appears normal without ulceration or discoloration.

A 68-year-old woman presented to our department with a chief complaint of swelling in the right mandibular gingiva. The patient had been aware of a mass in the right mandibular alveolar region for approximately 20 years. However, as there had been no noticeable increase in size, she did not seek medical attention. Recently, her primary dentist recommended further evaluation and treatment for the lesion, prompting her referral to our department. Intraoral examination revealed a soft, elastic mass in the buccal alveolar mucosa from the right canine to the first premolar region (Figure 1).

**Figure 2:**
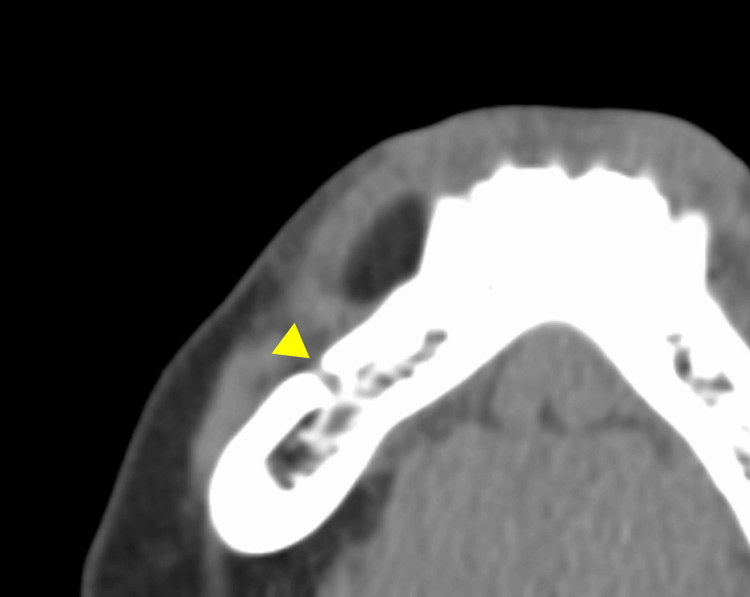
CT image Axial CT image showing a well-circumscribed, homogeneous low-density lesion measuring 17 × 7 × 10 mm in the right mandibular alveolar region. The lesion is located approximately 3 mm anterior to the mental foramen (arrow).

CT imaging demonstrated a well-circumscribed, homogeneous low-density lesion measuring 17 × 7 × 10 mm, located 3 mm anterior to the mental foramen (Figure 2).

**Figure 3:**
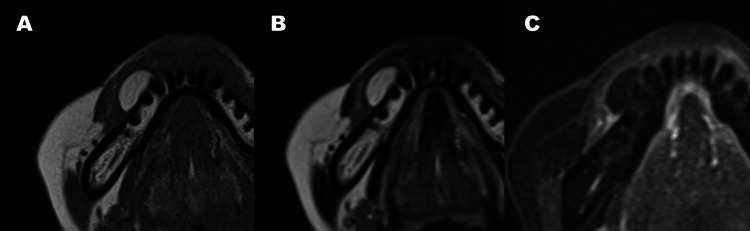
MRI findings T1-weighted image(A) and T2-weighted image(B) showing a homogeneous high-intensity signal corresponding to the mass. STIR image (C) showing a low signal intensity area consistent with fatty tissue characteristics.

MRI showed a high-signal intensity mass on T1- and T2-weighted images, with low intensity on short Tau inversion recovery (STIR), measuring 17 × 9 × 12 mm (Figure 3).

**Figure 4:**
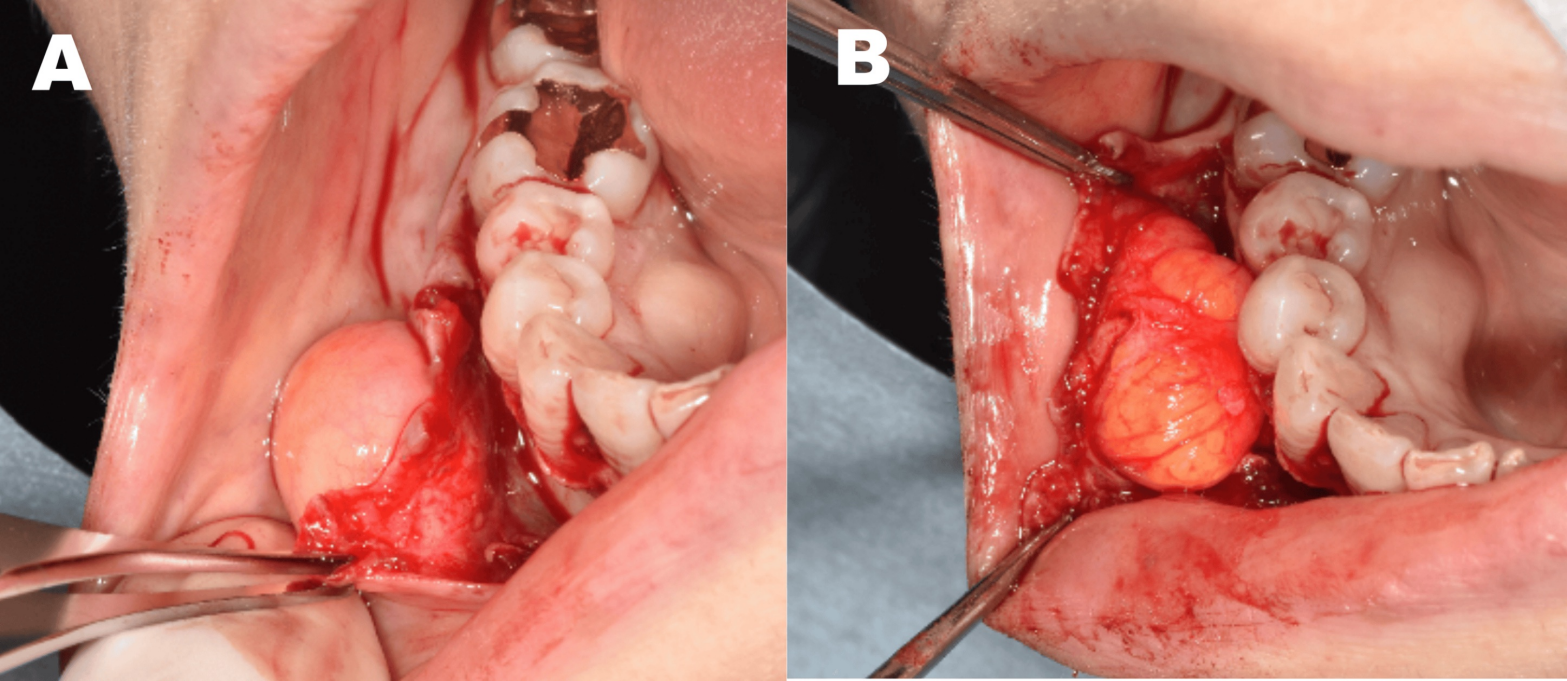
Intraoperative view of the surgical approach A triangular mucoperiosteal flap was elevated, preserving the overlying mucosa (A). The lipoma was accessed from the periosteal side and identified as a well-encapsulated mass prior to en bloc excision (B).

Based on these findings, a diagnosis of lipoma was made. Neither fine-needle aspiration nor ultrasonography was performed in this case, as the lesion exhibited long-term stability, a well-circumscribed margin, and imaging characteristics on CT and MRI that were highly suggestive of a lipomatous tumor. Under local anesthesia, a triangular mucoperiosteal flap was elevated with a vertical releasing incision mesial to the lateral incisor. The periosteum was carefully dissected to expose the encapsulated tumor without disturbing the overlying mucosa (Figure 4).

**Figure 5:**
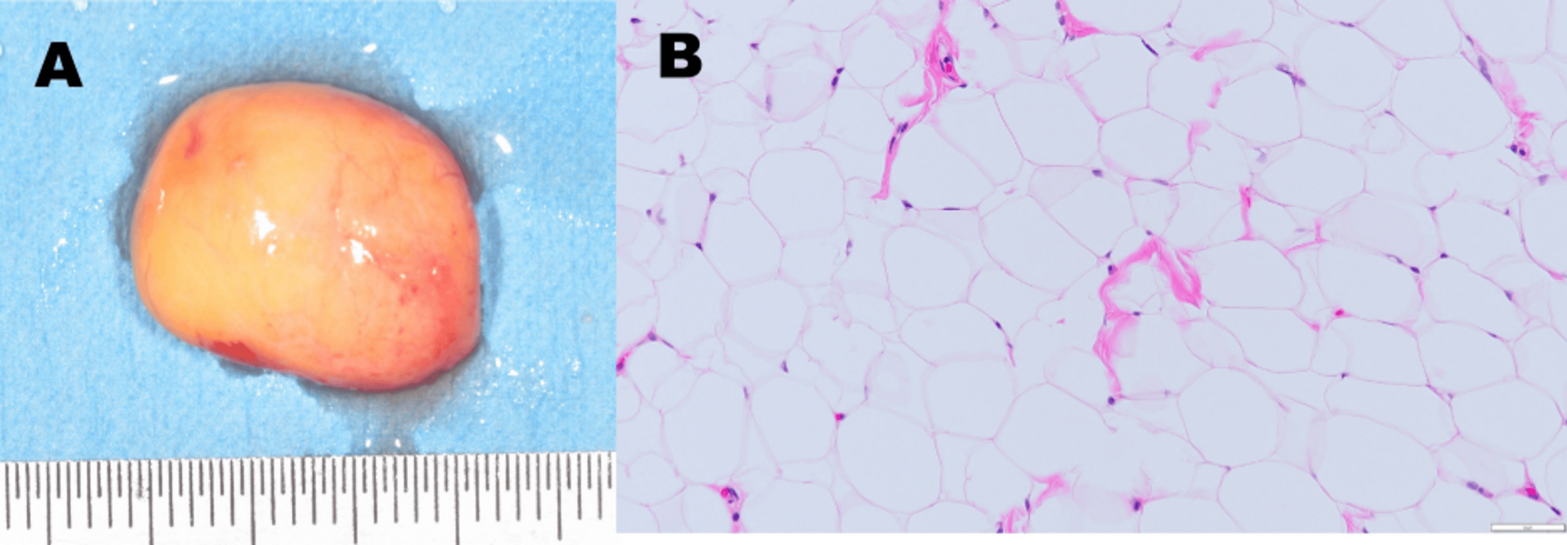
Resection specimen and histopathological findings The excised specimen shows a yellowish, soft, encapsulated mass consistent with a lipomatous lesion (A). Hematoxylin and eosin (H&E) staining reveals mature adipocytes with interspersed fibrous connective tissue, confirming the diagnosis of fibrolipoma (original magnification ×100) (B).

No adhesion to the mental nerve was observed. The lesion was removed en bloc via subperiosteal dissection. Histopathological diagnosis confirmed fibrolipoma (Figure 5). Gross examination revealed a well-encapsulated, yellowish soft mass. Histologically, the lesion consisted predominantly of mature adipocytes arranged in lobules. Upon closer inspection, thin fibrous septa were observed between lobules, although they were not prominent in the representative photomicrograph. These features supported a final diagnosis of fibrolipoma.

**Figure 6:**
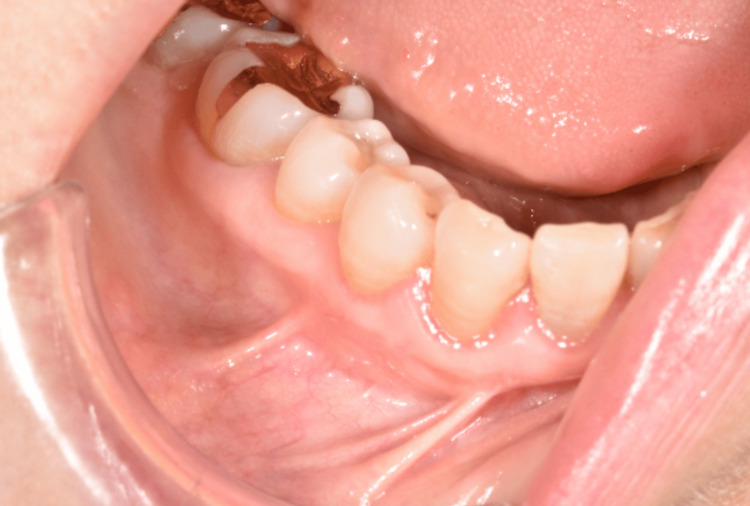
Postoperative intraoral photograph at follow-up Postoperative intraoral photograph at follow-up showing complete healing without scar formation or gingival recession in the surgical site.

The postoperative course was uneventful, with no sensory deficits or recurrence observed during the one-year follow-up period (Figure 6).

## Discussion

This case highlights two clinically important points. First, fibrolipomas involving the mandibular alveolar region, especially near the mental foramen, are extremely rare and represent a diagnostic and surgical challenge. Second, this case illustrates how a mucosa-sparing, subperiosteal surgical approach can allow for complete and safe tumor excision while preserving esthetics and function. Together, these findings emphasize the importance of individualized surgical planning and add to the growing body of literature supporting minimally invasive techniques for oral lipoma management.

In the present case, the lesion was soft and non-tender and had remained unchanged in size for approximately 20 years, without signs of rapid growth or discoloration. These features made vascular or neural tumors less likely. Combined with the well-defined margins and characteristic low-density and high-signal imaging on CT and MRI, a diagnosis of lipomatous tumor was strongly supported prior to surgical excision. In terms of prevalence, intraoral lipomas are most commonly found in the buccal mucosa, tongue, and lips [1,6,7]. Cases involving the mandibular alveolar region, such as ours, are seldom reported [2,4,5]. Several authors have described variations such as fibrolipoma and spindle cell lipoma arising in these rare locations, further complicating diagnosis [11]. While most lipomas present as painless, slow-growing masses, the differential diagnosis includes other benign entities such as neurofibromas, fibromas, and vascular lesions [3,8,12]. Therefore, preoperative imaging plays a pivotal role in characterization. The CT and MRI findings in our case - homogeneous low attenuation on CT and high T1/T2 signal with STIR suppression - are consistent with previous reports of lipoma [4,7,9].

Surgical management remains the cornerstone of treatment. In this case, the subperiosteal approach allowed us to avoid mucosal damage and protect the mental neurovascular bundle. This technique has been favorably described in recent literature for its esthetic and functional preservation, especially when used in conjunction with a triangular flap design [9,10]. Comparable approaches have been used in cases involving the tongue and buccal vestibule, demonstrating reduced morbidity and favorable healing outcomes [6,10]. Moreover, minimal manipulation of the overlying mucosa may reduce the risk of postoperative scarring, gingival recession, or altered sensation [7,11]. Although surgical excision remains the gold standard for treating intraoral lipomas, alternative techniques have been explored, especially for smaller or more superficial lesions. Minimally invasive options such as laser excision or electrosurgery have been reported to offer favorable healing, minimal scarring, and reduced operating time [13]. In this case, due to the lesion’s proximity to the mental foramen and its encapsulated nature, conventional subperiosteal excision was deemed most appropriate. However, future studies may explore the utility of such adjunctive techniques, particularly in cosmetically sensitive or high-risk anatomical regions. In cases where the lesion is larger, rapidly growing, or adherent to the surrounding neurovascular structures, preoperative considerations may include more detailed imaging, neurological mapping, or even multidisciplinary consultation. The choice of surgical approach must be tailored to balance complete excision with the preservation of nerve function and esthetics.

Histopathological examination confirmed the diagnosis of fibrolipoma - characterized by a lobulated mass of mature adipocytes interspersed with dense fibrous connective tissue [3,14]. Although the representative histological image primarily displays adipose tissue, the presence of thin fibrous septa within the lobulated structure - combined with the intraoperative finding of fibrous resistance during subperiosteal dissection - supports the diagnosis of fibrolipoma rather than a simple lipoma. Fibrolipomas are histologically characterized by mature fat cells interspersed with fibrous connective tissue, which can sometimes be subtle and easily overlooked in routine sections. This subtype, although benign, necessitates complete excision due to the potential for local recurrence, especially if incompletely resected [12,15]. Our surgical method ensured en bloc removal under direct visualization, minimizing the risk of recurrence. Literature reviews have shown recurrence rates for oral lipomas are low, particularly when complete excision is achieved [6,8,10]. Postoperative recovery in our patient was uneventful, with no signs of nerve disturbance or mucosal scarring. In this case, the patient was followed for one year postoperatively with no evidence of recurrence or sensory disturbance. While recurrence of fibrolipoma is rare when complete excision is achieved, long-term monitoring remains essential, especially in lesions involving critical anatomical landmarks.

## Conclusions

This case contributes to the limited number of documented intraoral fibrolipomas located near the mental foramen. It underscores the importance of preoperative imaging, careful differential diagnosis, and the value of a subperiosteal, mucosa-sparing technique in ensuring successful tumor removal while minimizing complications. The patient remained recurrence-free over a one-year follow-up period, with no sensory disturbances or mucosal scarring. This case reinforces the value of careful imaging evaluation, precise surgical planning, and technique selection in rare intraoral tumors. It contributes to the limited literature on fibrolipomas in sensitive oral regions and may aid clinicians in managing similar presentations with minimal morbidity. Future comparative studies or systematic reviews may help confirm the advantages of this approach in rare intraoral sites and refine the surgical protocols for oral soft tissue tumors.
